# Renal Arteriovenous (AV) Fistula after High-Grade Blunt Renal Trauma Caused by Traffic Accidents

**DOI:** 10.3390/jcm12196362

**Published:** 2023-10-04

**Authors:** Susanne Deininger, Peter Törzsök, Lukas Lusuardi, Sebastian Hubertus Markus Deininger, Thomas Freude, Florian Wichlas, Christian Deininger

**Affiliations:** 1 Department of Urology and Andrology, University Hospital Salzburg, Paracelsus Medical University, 5020 Salzburg, Austria; p.toerzsoek@salk.at (P.T.); l.lusuardi@salk.at (L.L.); 2 No Limit Surgery e.V. (NLS), 5020 Salzburg, Austria; s.deininger@hotmail.com (S.H.M.D.); f.wichlas@salk.at (F.W.);; 3 Department of Orthopedics and Traumatology, Salzburg University Hospital, Paracelsus Medical University, 5020 Salzburg, Austria; t.freude@salk.at

**Keywords:** kidney trauma, AV fistula, complication, coil embolization, blunt, laceration, arteriovenous malformation, vessel

## Abstract

Purpose: To report a series of three patients with traumatic renal AV fistulas after blunt renal laceration. Methods: We retrospectively analyzed the renal trauma cases treated in the Department of Urology of Salzburg University Clinic during a time period of 10 years concerning traumatic AV fistula formation and other clinical parameters. Results: In total, 3 cases of traumatic AV fistula formation were identified in 106 blunt renal trauma patients (2.8%), with a mean age of 39 (17–56) years. All renal traumas were classified as American Association for the Surgery of Trauma (AAST) grade IV. Two patients were primarily treated with ureteral stent; one was managed conservatively. All AV fistulas were diagnosed after a mean time of 7 (1–13) days. Two patients were symptomatic with gross hematuria, and the mean time between trauma and onset of symptoms was 11 (9–13) days. All cases were managed via coil embolization after a mean of 10 (8–13) days. Two patients received a second intervention after a mean of 18 (11–25) days. The mean AV fistula size was 18.7 (12–24) mm. Mean hemoglobin loss was 3.6 g/dL. One patient received one erythrocyte concentrate. Discharge was after a mean time of 13.3 (7–12) days, with the mean time of intensive care treatment being 2.3 (1–3) days. Conclusions: Traumatic renal AV fistula is a rare but severe complication associated with higher-grade renal trauma. It can become evident through hematuria or blood loss several days after the initial trauma. The availability of coil embolization in a trauma center can help kidney preservation management.

## 1. Introduction 

Renal laceration with 4–10% of all abdominal trauma cases is one of the most common intra-abdominal injuries in Western countries, mainly in the context of blunt force trauma [1,2,3]. Renal traumata may manifest in isolation or concomitantly with injuries to other thoraco-abdominal organs (such as the spleen and liver) or rib fractures [4]. Blunt trauma is the leading cause of injury in up to 90% of cases [5], while penetrating traumas like stab wounds or gunshots are seldom encountered in the Western world. Individuals who experience renal lacerations tend to be on the younger side, with an average age of approximately 30.8 years, and the majority, accounting for 72%, are male [6]. Renal trauma can result from a variety of blunt trauma mechanisms, including incidents such as motor vehicle accidents, minor falls, participation in sports, and pedestrian collisions. In a study conducted by Hurtuk et al., which analyzed almost 16,000 cases of renal trauma in the United States (comprising both blunt and penetrating injuries), it was found that 73% of all cases were attributed to traffic accidents, 19% to assaults, and 8% to various other causes like blows, firearm incidents, suicides, machinery accidents, and piercing injuries [7]. In another study by Voelzke et al., which focused on over 10,000 cases of blunt renal trauma, the most common mechanisms of injury were traffic accidents (63%), followed by falls (14%) and participation in sports activities (11%) [6]. 

The guideline for urological trauma issued by the European Association of Urology (EAU) recommends primary non-operative management (NOM). This approach encompasses endourological procedures like the placement of a double-J stent, the use of drainage, or interventional radiology techniques such as selective angioembolization for the treatment of renal trauma [8]. The application of NOM demonstrates a significant clinical advantage for patients compared to the surgical approach; Mingoli A. et al. showed in a meta-analysis of over 13,000 renal trauma cases that NOM, regardless of the American Association for the Surgery of Trauma (AAST) grades, resulted in lower mortality and morbidity rates (each * *p*< 0.001) [9]. 

But organ-preserving procedures enable the occurrence of sometimes life-threatening complications on the kidney itself, such as urine leak or fistula, abscess, vascular thrombosis, bleeding, renal infarction, renal dysfunction, and the formation of pseudoaneurysm or arteriovenous (AV) fistula [10]. The AV fistula is an abnormal connection between artery and vein and can occur in different body regions. Beneath trauma, other causes of AV fistula formation have been described: idiopathic, congenital, or iatrogenic.

The data on traumatic arteriovenous fistulas are limited, and the prevalence remains unclear. Among the 889 renal trauma cases analyzed by Starnes et al. in 2010 with 65.5% penetrating and 34.5% blunt renal traumas, only 1 AV fistula case was reported (0.11%) [10]. Research on arteriovenous fistulas (AV fistulas) is challenging due to their low incidence. In a cohort of 9500 arteriographic studies of the kidneys, Cho et al. reported an incidence of 0.04% for congenital AV fistulas [11]. In the general population, this incidence is likely to be even lower. The following article analyzes three cases of blunt trauma-related renal AV fistulas treated in the Department of Urology and Andrology, Salzburg University Hospital. 

## 2. Materials and Methods

The study was a collaborative effort involving the Department of Urology and Andrology, along with the Department of Orthopedics and Traumatology, University Hospital Salzburg, Paracelsus Medical University, 5020 Salzburg, Austria. The University Hospital represents a Level 1 Trauma Center, and it not only cares for trauma patients from Salzburg but also extends its services to individuals with severe injuries referred from nearby smaller hospitals (Level II and III trauma centers). Consequently, there is a notable influx of renal trauma cases that necessitate medical attention and further assessment. The study involved a retrospective analysis of data from patients who experienced blunt renal trauma between January 2010 and March 2020. Approval from the ethics commission of the Province of Salzburg, Austria was obtained, with the reference number being 1078/2020 (meeting of 27 May 2020). No cases with penetrating kidney trauma or cases treated under the supervision of another clinic due to severe injuries or polytrauma were included. In total, 106 patients with blunt renal trauma were accompanied during that time period. Among these 106 cases of renal trauma, there were notably 3 cases of traumatic AV fistula formation (2.8%). Due to this, this research group has chosen to present these cases. We registered gender, age, trauma mechanism, concomitant injuries, therapy, transfusion rate, and time of inpatient and intensive care treatment in these patients. 

## 3. Results

An overview of the patients’ characteristics and findings can be found in Table 1.

### 3.1. Patient I: Male Patient, Aged 17 Years

**Figure 1:**
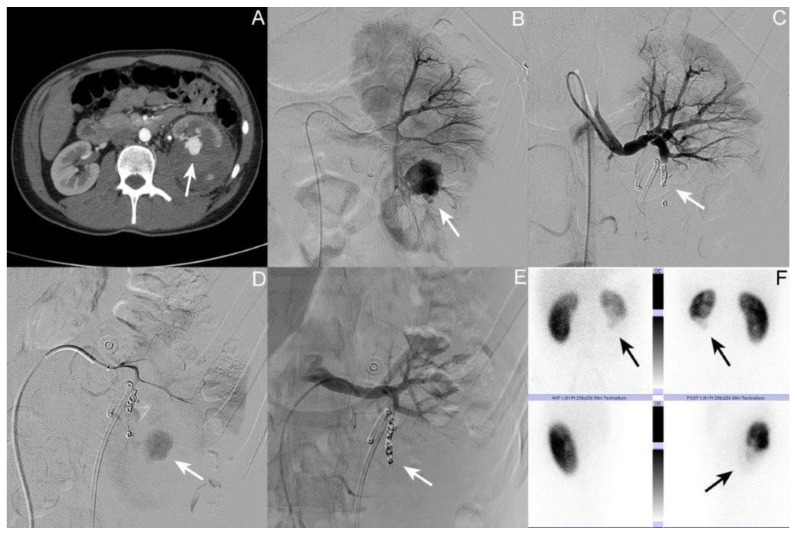
Selected imaging of case I. (**A**) Axial contrast-enhanced computed tomography (CT) scan of the abdomen; the arrow shows the 24 mm arteriovenous (AV) fistula at the lower pole of the left kidney. (**B**) Angiography of the left kidney; the arrow shows the AV fistula. (**C**) Angiography post coil embolization; the arrow shows the coils in situ in the lower pole segmental artery of the left kidney. (**D**) Repeat angiography showing persistent AV fistula (arrow). (**E**) Angiography post second coil embolization; the arrow shows the coils in situ in the lower pole segmental artery of the left kidney. (**F**) Technetium-99 m dimercaptosuccinic acid (DMSA) renal scintigraphy showing no tracer accumulation in the lower kidney pole on the left side (arrow); contribution right/left kidney 65%:35%.

Representative excerpts from the CT scans, radiographic evaluation, and DMSA (dimercaptosuccinic acid) renal scintigraphy performed can be found in Figure 1. The patient was admitted at the age of 17 after a motorcycle accident. The first whole-body CT scan during shock-room management showed a grade IV renal injury according to AAST on the left side with laceration of the lower calices and consequently perirenal urinoma and hematoma. No concomitant injuries were detected. Their creatinine level at admission was 0.9 mg/dL, with a hemoglobin (Hb) value of 14.4 g/dL. On the same day, the insertion of a Mono-J (MJ) ureteral stent was performed to drain the renal pelvis. The patient was admitted to the intensive care unit for one day and then transferred to the normal ward in a stable clinical condition. A routinely performed control CT after two days showed stable urinoma and hematoma formation. The MJ was changed to a Double-J (DJ) ureteral stent. During hospitalization, the patient was in a stable clinical condition and was discharged after seven days. Thirteen days after the initial trauma, the patient presented again in our outpatient ambulance due to flank pain on the left side and gross hematuria. Their creatinine level at admission was 0.9 mg/dL, with an Hb value of 10.2 g/dL. A repeat CT scan of the abdomen showed large perirenal hematoma on the left side and a 24 mm AV fistula of the lower pole of the left kidney (Figure 1A). A coil embolization of the lower kidney pole segmental artery was conducted the same day (Figure 1B,C). The patient was discharged again after five days in a stable clinical condition with an Hb value of 9.8 mg/dL. Another seven days later, he was readmitted due to gross hematuria, with the repeat angiography showing persistent AV fistula (Figure 1D). Another coil embolization of the lower pole segmental artery of the left kidney was performed (Figure 1E). Four days later, he was discharged again. After eleven days, the patient needed another inpatient stay due to febrile urinary tract infection (UTI) requiring a change in the indwelling ureteral stent and antibiotic therapy. Sixty-one days later, the DJ stent was removed in an ambulant setting. Two hundred and forty-eight days after the initial trauma, a Technetium-99 m DMSA renal scintigraphy showed no tracer accumulation in the lower kidney pole on the left side, with contribution of the right/left kidney of 65%:35% (Figure 1F). Their creatinine level at that time was 1.2 mg/dL. 

### 3.2. Patient II: Male Patient, Aged 44 Years 

**Figure 2:**
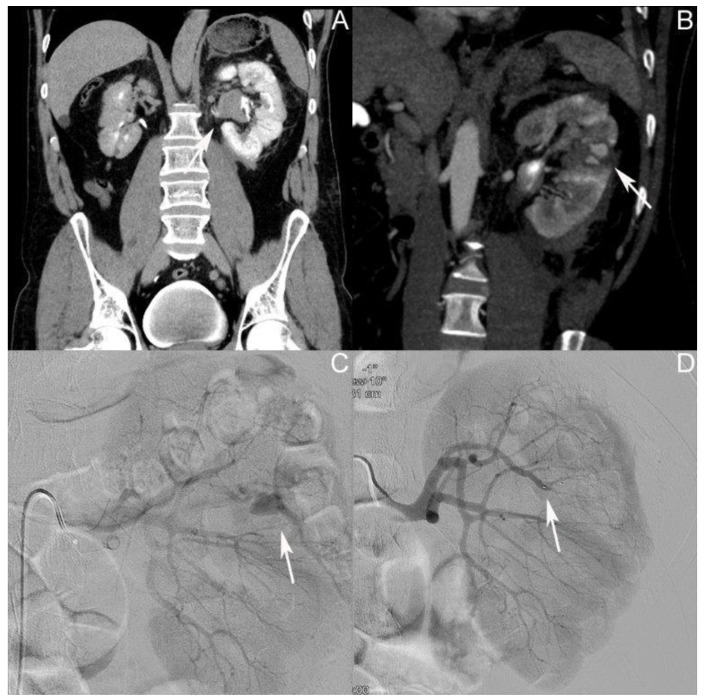
Selected imaging of case II. (**A**) Coronary contrast-enhanced computed tomography (CT) scan of the abdomen; the arrow shows the affected left kidney with coagula in the renal pelvis. (**B**) Coronary contrast-enhanced CT scan of the abdomen; the arrow shows the 15 mm arteriovenous (AV) fistula in the middle pole of the left kidney. (**C**) Angiography of the left kidney; the arrow shows the AV fistula. (**D**) Angiography post coil embolization; the arrow shows the coils in situ.

Representative excerpts from the CT scans, radiographic evaluation, and DMSA renal scintigraphy performed can be found in Figure 2. The patient was admitted at the age of 44 after a bike accident. The first whole-body CT scan during shock-room management showed a grade IV renal injury according to AAST on the left side, with perirenal hematoma 25 mm in diameter, and coagula in the renal pelvis and urinary bladder (Figure 2A). No concomitant injuries were detected. Their creatinine level at admission was 1 mg/dL, with an Hb value of 13.5 g/dL. The patient was admitted to the intensive care unit. The first control CT was performed after one day, showing a 12 mm AV fistula in the middle pole of the left kidney. Conservative treatment was continued. Three days after the initial trauma, the patient was transferred to the normal ward in a stable clinical condition. On day nine after the initial trauma, the patient developed gross hematuria and rising inflammatory values; the CT showed an AV fistula in the middle pole of the left kidney (Figure 2B). Coil embolization of the AV fistula was performed on the same day (Figure 2C,D). The patient received one erythrocyte concentrate. Twelve days after the initial trauma, the patient was discharged with an Hb value of 8.1 g/dL and a creatinine level of 0.8 mg/dL in a stable clinical condition.

### 3.3. Patient III: Female Patient, Aged 56 Years

**Figure 3:**
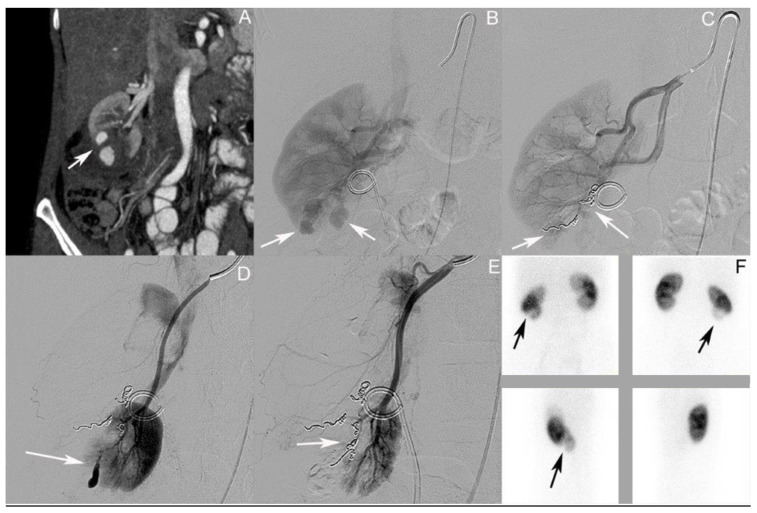
Selected imaging of case III. (**A**) Coronary contrast-enhanced computed tomography (CT) scan of the abdomen; the arrow shows several up to 20 mm in size AV fistulas/pseudoaneurysms in the ruptured parenchyma of the lower pole of the right kidney. (**B**) Angiography of the right kidney with Double-J ureteral stent in situ; the arrow shows the AV fistulas. (**C**) Angiography post coil embolization; the arrow shows the coils in situ in the lower pole of the right kidney. (**D**) Repeat angiography showing persistent AV fistula (arrow) post second coil embolization; the arrow shows the coils in situ in the lower pole of the left kidney. (**E**) Angiography post second coil embolization; the arrow shows the coils in situ in the lower pole of the right kidney. (**F**) Technetium-99 m dimercaptosuccinic acid (DMSA) renal scintigraphy showing no tracer accumulation in the lower kidney pole on the right side (arrow); contribution left/right kidney of 61%:39%.

Selected imaging of this case can be found in Figure 3. The patient was admitted at the age of 56 after a bike accident. The first whole-body CT scan during shock-room management showed a grade IV renal injury according to AAST on the right side, with a perirenal hematoma 25 mm in diameter, a perirenal urinoma, and coagula in the urinary bladder. No concomitant injuries were detected. Their creatinine level at admission was 1.05 mg/dL, with an Hb value of 12.9 g/dL. An MJ ureteral stent was positioned the same day, and the patient was admitted to the intensive care unit. After three days, the patient was transferred to the normal ward as they were in a stable clinical condition. A change from an MJ to DJ ureteral stent was performed on day four. On day seven, the first control CT was performed, showing several AV fistulas/pseudoaneurysms up to 20 mm in size in the ruptured parenchyma of the lower pole of the right kidney (Figure 3A). The day after, coil embolization was performed without complications (Figure 3B,C). On day eleven, another control CT showed a persistent AV fistula of 15 mm, which was again treated using coil embolization (Figure 3D,E). On day 21, after the initial trauma and 1 day after the re-embolization, the patient was discharged in a stable clinical condition. Their Hb value was 9.1 g/dL and their creatinine level was 0.75 mg/dL at that time. The DJ stent was removed in an ambulant setting. One hundred and six days after the initial trauma, a Technetium-99 m DMSA renal scintigraphy showed no tracer accumulation in the lower kidney pole on the right side, with a contribution of the left/right kidney of 61%:39% (Figure 3F). Their creatinine level at that time was 0.78 mg/dL. 

## 4. Discussion

The literature about AV fistulas in general is heterogeneous, concerning different body regions. Injuries resulting in AV fistulas within the kidney can be attributed to either penetrating or blunt trauma, percutaneous or open biopsy, or surgical procedures. Iatrogenic injury, particularly percutaneous renal biopsy, is one of the leading causes of traumatic renal AV fistulas. The literature reports of an incidence of 7.4–11% following this procedure [12]. Another potential cause of renal AV fistula is nephron-sparing surgical procedures. In 2011, Hyams et al. analyzed 998 patients after minimally invasive partial nephrectomy and found 2% operation-related pseudoaneurysms and AV fistulas [13]. 

And the risk of vascular injuries increases with specific surgical techniques. For instance, Albani et al. in 2003 demonstrated a higher risk of arterial pseudoaneurysm after minimally invasive partial nephrectomies compared to open surgical approaches [14]. Tufano et al. reported an AV fistula following robotic partial nephrectomy in a 65-year-old patient [15]. The tumor’s location also appears to play a role in the formation of vascular malformations. For instance, Omae et al. in 2015 demonstrated a higher risk of developing arterial pseudoaneurysms with larger tumors (*p* = 0.02), with greater proximity of the tumor to the collecting system or sinus (i.e., a higher N component from the R.E.N.A.L. nephrometry scoring system, *p* = 0.01), and with a higher incidence of intraoperative sinus exposure or opening of the collecting system (*p* < 0.01) [16]. Also, after stone management via percutaneous nephrolithotomy (PCNL), AV fistulas have been described. The literature reports an incidence of 0.3 up to 8.3% [17,18], with previous ipsilateral renal surgery, higher stone complexity, injury to the renal pelvis, and multiple access tracts further increasing risk [19]. 

One fact applies to all AV fistulas: the onset of symptoms in most cases is delayed and can occur up to years after the initial trauma [20,21]. The literature discusses the time leak between trauma and occurrence of the fistula being caused by continuous enlargement of an initially small fistula or by clot resolution that covered the fistula opening in the beginning [20]. The onset of symptoms in our patient population was after a mean time of eleven days, which is comparable to the findings of other research groups. In the patient population of iatrogenic vascular lesions (pseudoaneurysm and AV fistula), after minimally invasive partial nephrectomy analyzed by Hyams et al. in 2011, the mean time of the onset of symptoms was 14.5 days after the intervention [13]. 

Certainly, AV fistulas can indeed cause a wide range of clinical symptoms, and the exact presentation can vary depending on the location, size, and cause of the fistula; hematuria, pain, retroperitoneal bleeding, swelling, high- output heart failure, anemia, vascular bruits, or hypertension can all be possible symptoms of AV fistulas [12,22,23]. But AV fistulas may also be asymptomatic; in a retrospective data analysis of 69 AV fistulas in various body regions performed by Kollmeyer et al. in 1981, about half of these vessel malformations were incidental findings [24].

Following kidney surgery or trauma, hematuria can indeed serve as an initial warning sign of the development of an AV fistula. In our patient group, two out of three patients suffered from gross hematuria, which is also the most commonly reported symptom in the literature [13]. This is an important point to consider, as patients who experience hematuria after such procedures should be evaluated promptly for the possibility of an AV fistula, which can have significant implications for their health and may require further medical intervention. More seldom symptoms like heart failure due to cardiopulmonary overload [20] or secondary hypertension through the activation of renin–angiotensin–aldosterone system (RAAS) [25] have been described. 

In our patient population, two out of three cases of AV fistula were initially only detected in a control CT imaging not on the basis of symptoms. The gold standard in the diagnosis of renal AV fistula is contrast-enhanced computed tomography (CT) [26,27]. This diagnostic method excels at precisely delineating the anatomy of the fistula, assessing its impact on nearby structures, and aiding in the development of an appropriate treatment plan. The non-contrast CT is only capable of revealing a perirenal hematoma [28]. Still the color Doppler ultrasound is well suited to detect a renal AV fistula [29]; phenomena such as a region of high-velocity shift and a low-resistive index can be indicative of AV fistulas [30]. 

In our collective, one of two patients where the AV fistula was an incidental finding was treated conservatively at first (case II). Only after the development of symptoms (gross hematuria and rising inflammatory parameters) was the AV fistula managed via coil embolization. Just in one case was the AV fistula discovered after the development of symptoms (Case I: flank pain and gross hematuria). Case I is also the case in which the diagnosis of the AV fistula within 13 days after the trauma was made last. 

The spontaneous closure of AV fistulas in general has been described in the literature [31,32] and may occur in renal trauma patients likewise. In the study of Hyams et al. from 2011, 2 out of the 20 cases of AV fistula or pseudoaneurysm did not require therapy due to spontaneous resolution [13].

In our patient population, all cases were treated with radiographic embolization [33]. Small studies have shown good success rates of coil embolization in renal AV fistulas [34] with easily controllable complications. Potential risks of coil embolization of renal AV fistula include bleeding, vascular damage, pulmonary embolism or stroke, as well as fistula recurrence. Eighty-six percent of the patients treated via coil embolization for renal angiomyolipoma studied by Wang C. et al. in 2017 suffered from post-embolization syndrome (fever, nausea/vomiting, and abdominal pain). All of them could be treated conservatively [35]. In cases of greater or high-flow AV fistulas, the risk of the migration of embolic material in the pulmonary vessels can be anticipated using an AMPLATZER™ Vascular Plug [36]. 

In the context of challenging clinical scenarios where coil embolization fails to achieve the desired outcome or in situations where the immediate availability of interventional radiology services is lacking within the trauma center, nephrectomy emerges as a viable therapeutic option [37]. This surgical intervention seems to be only ultima ratio nowadays. 

## 5. Conclusions 

Renal AV fistula with 2.8% in our patient population is a rare complication after blunt renal trauma. Iatrogenic renal AV fistulas are more commonly encountered, for example, in association with renal biopsies or after nephron-sparing surgeries. Symptoms such as flank pain or hematuria can serve as potential warning signs for the development of an AV fistula, although patients may also remain asymptomatic for an extended period. If symptoms do occur, they can typically be expected more than 10 days after the initial trauma. Contrast-enhanced CT scans represent the gold standard in diagnosing AV fistulas; however, color Doppler ultrasound can also be a helpful diagnostic tool. The conservative management of renal AV fistulas appears feasible, but intervention may be necessary in cases of significant blood loss or symptomatic presentation. In our cohort, all fistulas were successfully treated using coil embolization, with nephrectomy considered as a last resort, especially in the absence of interventional radiology resources.

## Figures and Tables

**Table 1:** Patient characteristics and findings. (AAST: American Association for the Surgery of Trauma; AV: arteriovenous; DMSA: dimercaptosuccinic acid; HU: hematuria; Hb: hemoglobin value; IP: inflammatory parameters; mm: millimeter; NA: not applicable; TA: tracer accumulation; US: ureteral stent; UTI: urinary tract infection).

	Case I	Case II	Case III	Mean if Applicable
Age at trauma	17	44	56	39
Gender	Male	Male	Female	
Trauma mechanism	Motorcycle accident	Bike accident	Bike accident	
Concomitant injuries	None	None	None	
AAST grade of renal trauma	IV	IV	IV	
Side of renal trauma	Left	Left	Right	
Primary treatment	Placement of US	Conservative	Placement of US	
Time of first diagnosis of AV fistula after the initial trauma in days	13	1	7	7
Size of AV fistula in mm	24	12	20	18.7
Time between trauma and onset of symptoms in days	13	9	NA	11
Symptoms	Flank pain, HU	HU, IP	None	
Number of coil embolizations performed	2	1	2	1.7
Time of coil embolization(s) in days after the trauma	13 25	9	8 11	10 18
Time of intensive care treatment in days	1	3	3	2.3
Time of inpatient treatment in days	16	12	12	13.3
Hb loss in g/dL	4	5.4	1.4	3.6
Number of erythrocyte concentrates given	0	1	0	0.3
Creatinine change in mg/dL	+0.3	−0.2	−0.27	
Complications	HU, UTI	HU, need for transfusion, IP		
Time of DMSA renal scintigraphy after the trauma in days	248	NA	106	177
Result of DMSA renal scintigraphy	-No TA in the lower kidney pole on the left side -Contribution: right/left kidney 65%:35%	NA	-No TA in the lower kidney pole on the right side -Contribution: left/right kidney 61%:39%	

## Data Availability

The data presented in this study are available on request from the corresponding author.
